# Reader’s digest version of scientific writing: comparative evaluation of summarization capacity between large language models and medical students in analyzing scientific writing in sleep medicine

**DOI:** 10.3389/frai.2024.1477535

**Published:** 2024-12-24

**Authors:** Jacob Matalon, August Spurzem, Sana Ahsan, Elizabeth White, Ronik Kothari, Madhu Varma

**Affiliations:** ^1^Medical school, California University of Science and Medicine, Colton, CA, United States; ^2^Department of Medical Education and Clinical Skills, California University of Science and Medicine, Colton, CA, United States

**Keywords:** sleep medicine, scientific writing, artificial intelligence, natural language processing, large language models, medical education, medical students

## Abstract

**Introduction:**

As artificial intelligence systems like large language models (LLM) and natural language processing advance, the need to evaluate their utility within medicine and medical education grows. As medical research publications continue to grow exponentially, AI systems offer valuable opportunities to condense and synthesize information, especially in underrepresented areas such as Sleep Medicine. The present study aims to compare summarization capacity between LLM generated summaries of sleep medicine research article abstracts, to summaries generated by Medical Student (humans) and to evaluate if the research content, and literary readability summarized is retained comparably.

**Methods:**

A collection of three AI-generated and human-generated summaries of sleep medicine research article abstracts were shared with 19 study participants (medical students) attending a sleep medicine conference. Participants were blind as to which summary was human or LLM generated. After reading both human and AI-generated research summaries participants completed a 1–5 Likert scale survey on the readability of the extracted writings. Participants also answered article-specific multiple-choice questions evaluating their comprehension of the summaries, as a representation of the quality of content retained by the AI-generated summaries.

**Results:**

An independent sample t-test between the AI-generated and human-generated summaries comprehension by study participants revealed no significant difference between the Likert readability ratings (*p* = 0.702). A chi-squared test of proportions revealed no significant association (*χ*^2^ = 1.485, *p* = 0.223), and a McNemar test revealed no significant association between summary type and the proportion of correct responses to the comprehension multiple choice questions (*p* = 0.289).

**Discussion:**

Some limitations in this study were a small number of participants and user bias. Participants attended at a sleep conference and study summaries were all from sleep medicine journals. Lastly the summaries did not include graphs, numbers, and pictures, and thus were limited in material extraction. While the present analysis did not demonstrate a significant difference among the readability and content quality between the AI and human-generated summaries, limitations in the present study indicate that more research is needed to objectively measure, and further define strengths and weaknesses of AI models in condensing medical literature into efficient and accurate summaries.

## Introduction

The integration of artificial intelligence (AI) systems like large language models (LLM) and natural language processing (NLP) into healthcare and medical education is a rapidly growing field, with significant advancements and applications already in practice. Kaul et al. (2020) described many clinical applications of AI systems already approved by the FDA in multiple fields like radiology, cardiology, dermatology, and gastroenterology. Additionally, a systematic review by Abbasgholizadeh Rahimi et al. (2021) described how AI models currently are experimentally used for diagnosis, detection, and surveillance purposes in different healthcare systems, and how particular benefits may exist in the implementation within community-based primary health care settings. This review also highlighted marked variability and a lack of consistency in the implementation of different AI methods, analysis techniques, and outcomes of AI implementation. Li et al. (2022) also highlights the use of NLP AI models for summarizing unstructured data within electronic medical records (EMR), indicating utility for NLP models in processing medical information to generate medical writing. Despite current applications developing and already being utilized within healthcare, Parisis (2019) reported a lack of awareness in 40% of scientists regarding the potential of AI to be integrated into healthcare systems, and that opinions on the use of AI range from panic to strong optimism. In addition to diagnosis and data management within clinical medicine and EMR systems, the utilities of AI language systems in medical writing, medical education, and public health are vast.

As another example of LLMs being used to summarize medical information and generate medical writing, the OpenEvidence Application Programing Interface (API) is one of the first publicly available applications. The OpenEvidence API was the first AI systems to score over 90% on a United States Medical Licensing Exam, outperforming other nonspecific and medicine-specific AI systems. OpenEvidence also offers a publicly available LLM application, where one can ask medical questions and receive summarized, written medical information as a sort of medical-specific search engine (OpenEvidence, 2023). While OpenEvidence and some other LLMs provide citations for summarized medical literature, enabling the reader to fact-check, AI-generated summaries of medical literature for public use or use within medical education still present risks in producing and propagating incorrect information, or misinterpreting data. Similarly, the use of NLP models to aggregate EMR data may have great risks for patients, providers, and healthcare systems (Liao et al., 2023). The present study aims to address some of these risks by assessing qualities of AI-generated medical writing, with consideration to literary readability and the quality of content maintained after a LLM summarizes published medical research. Evaluating this type of content allows for the determination of the effectiveness and reliability of written AI-generated medical summaries, as variability or issues with AI-generated writing may have impactful implications for public health literacy, medical education, and medical practice.

Specifically, the present study intends to evaluate the ability of a LLM to collate peer-reviewed sleep medicine research articles into digestible, educational summaries at the medical school level, without sacrificing meaningful content. Additionally, we will compare the readability and content quality of AI-generated summaries with equivalent summaries produced by medical students. This comparison allows for assessing the comprehensibility of AI-generated summaries relative to those made by medical students, thereby testing the null hypothesis that no significant difference in comprehensibility exists between the two. If the capabilities of generative AI tools and LLMs could be deemed acceptable, without losing meaningful content, then the tools could be used to create scalable forms of a reader’s digests, which could be distributed to medical students, physicians, and specific patient populations. This could prove beneficial within medical education, by increasing exposure to emerging medical research, particularly in medical domains that may commonly be neglected, like sleep medicine.

## Methods

The study was approved by the California University of Science and Medicine (CUSM) Institutional Review Board under the protocol number HS-2023-14. The present study aims to compare the subjective readability of summaries of research article abstracts generated by AI software to the readability of summaries of the same abstracts generated by humans (Medical Students). This study also aims to compare the quality of information retained between summary type, AI-generated and human-generated, by having medical students answer multiple choice questions after reading the summaries.

### Participant recruitment

A non-probability convenience sampling method was used to gather participants for this research due to cost and practicality. Participants were recruited from the attendees at a Sleep Medicine conference at the California University of Science and Medicine (CUSM) medical school.

To recruit participants, the study researchers verbally explained the study to the audience members at the conference. Willing participants raised their hand and were given a sign-up form, printed consent forms, and assigned a unique identification number. Nineteen participants signed up and were randomly assigned into group A and group B. Group A consisted of ten participants and group B consisted of nine participants.

### Materials

Group A completed Form A of the study and group B completed Form B. These forms were created by study researchers in Google Forms and QR codes were given to participants. Both Form A and Form B consisted of the same three sleep medicine research article abstract summaries chosen by the study team to be of comparable difficulty.

Summaries of abstracts of three sleep medicine articles were generated by both the Generative Pre-trained Transformer (GPT) GPT-3.5-turbo API algorithm, within OpenAI’s free application ChatGPT, and by a consistent study investigator performing this study. The study investigator making the human-generated summaries remained consistent for all three articles to eliminate bias across the article summaries. GPT-3.5-turbo was accessed in March of 2023.

Each form contained one human-generated summary and two AI-generated summaries. The order of the three summaries within Form A and Form B was also kept equal, but the order of the summary type in each form varied. Specifically, Form A contained a human generated summary first, followed by two AI-generated summaries. Form B contained two AI-generated summaries, followed by the human generated summary. Regardless of summary type being AI or human generated, the length of the summaries was similar across each individual article. For example, the first summary contained 142 words in Form A and 122 words in Form B. The second summary contained 72 words in both forms, and the third summary contained 215 words in Form A and 269 words in Form B.

### Procedure

Participants were told that they would be reading and rating sleep medicine article summaries of abstracts generated by either MD, DO, or PA students in American medical programs, and that the summary samples could be from each of these student types or none of them. This aimed to blind the participants to the use of AI-generated summaries, and to make participants think they would be rating the summaries based solely on the authors’ medical degrees.

Despite the use of participant deception, the goal of the study required blinding participants to the use of AI summaries to prevent subject bias, and the CUSM Institutional Review Board approved this process and the use of human subjects with proper ethical considerations. Additionally, all subject data was de-identified of any personal information, and no academic or restricted information was collected during the study.

Participants were instructed to read each article one at a time, and after each article, participants indicated readiness to continue before answering the following question, “On a scale of 1–5, rate the article on readability.” In this Likert scale, a 1 represented “incomprehensible, did not understand,” and a 5 represented “brilliantly worded, easy to read and comprehend.” From these Likert scale answers, we collected 38 total ratings in response to AI summaries and 19 ratings in response to human summaries. Lastly, the two forms asked participants to answer three multiple choice questions per summary, to gauge comprehension of the summaries based on content. Form A and Form B contained the exact same comprehension questions with defined correct answers, with the same answer options ranging from A to F. The order of the comprehension questions in each form remained equal, and the answer options remained in the same order as well. Collectively, with 19 participants answering nine comprehension questions per assigned form, we collected 171 total responses for the comprehension questions. Of the 171 answers, we collected 114 responses from AI summaries, and 57 responses from human summaries.

### Statistical analysis

The Likert scale readability ratings between AI and human generated summaries were analyzed in SPSS with an independent samples T-test. The comprehension questions were graded for correctness, and the frequencies of correct versus incorrect answers between AI and human generated summary types were analyzed through a 2×2 cross-tabulation table in SPSS to obtain both a McNemar (paired chi-squared) test result and a chi-squared test of independence result. This comprehension question analysis specifically tested our null hypothesis of there being no difference in the frequency of correct responses between AI and human generated summary types, as a measure of comprehensibility and content quality. The 2×2 cross-tabulation table can be found in Table 1.

**Table 1:** Cross-tabulation data for the correct and incorrect comprehension test results between AI and human summary groups.

2×2 Cross-tabulation data				
Comprehension test result frequencies				
		Correct	Incorrect	Total
Summary type	AI	73	41	114
	Human	31	26	57
Total		104	67	171

## Results

**Figure 1:**
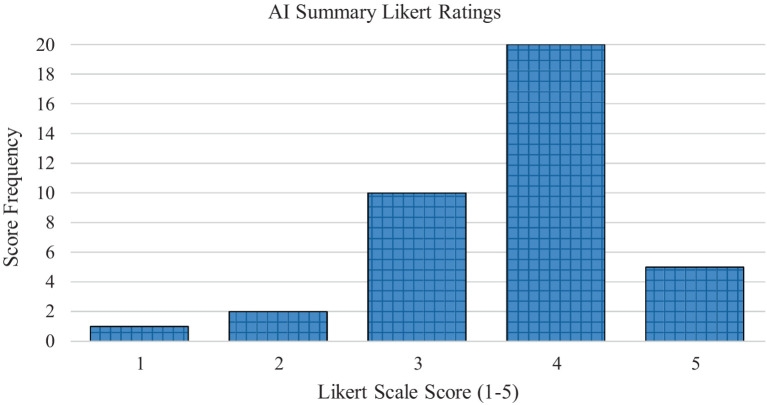
Average Likert scale readability ratings for the AI-generated summary group.

**Figure 2:**
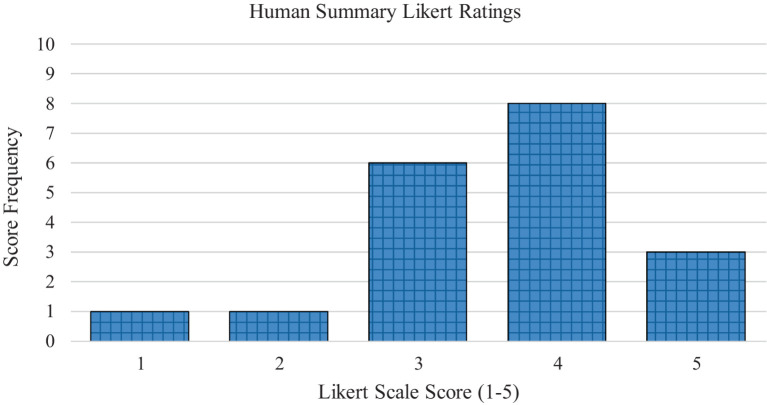
Average Likert scale readability ratings for the human generated summary group.

In the AI-generated summary group, the article summaries had a mean readability score on the 1–5 Likert scale of 3.68 (*SD* = 0.87). In the human generated summary group, the article summaries had a mean readability score of 3.58 (*SD* = 1.02). Figure 1 displays the frequencies of the Likert scale ratings for the AI-generated summaries. Figure 2 displays the frequencies of the Likert scale ratings for the human generated summaries. Table 2 displays the descriptive frequency statistics for both the AI-generated and human generated groups.

**Table 2:** Descriptive frequency statistical for the Likert scale readability ratings.

Descriptive statistics		
	AI summaries	Human summaries
N (valid)	38	19
N (missing)	0	19
Mean	3.6842	3.5789
Median	4.00	4.00
Mode	4.00	4.00
Std. deviation	0.87318	1.01739
Variance	0.762	1.035
Skewness	−0.860	−0.769

Based on the 57 Likert scale ratings, the independent samples T-test reveals no significant difference between the readability of the AI-generated and human generated summaries, yielding a nonsignificant result at the 5% significance level (*p* = 0.702). The independent samples T-test statistical results can be seen in Table 3. This comparison gives insight into the literary readability of the two summary types being comparable, as indicated by a body of primarily medical students.

**Table 3:** Independent *t*-test results between the mean AI-generated and human generated summary Likert scale readability ratings.

Independent samples *t*-test							
			Significance		Mean difference	95% CI	
	*t*	df	One-sided *p*	Two-sided *p*		Lower	Upper
Equal variances assumed	0.406	55	0.343	0.686	0.10526	−0.41439	0.62492
Equal Variances not assumed	0.386	31.6	0.351	0.702	0.10526	−0.45114	0.66166

Based on the 171 multiple choice responses on comprehension, the cross-tabulation analyses reveal no significant difference between the proportion of correct answers between summary types, thereby failing to reject the null hypothesis of the comprehension test scores being independent from the AI and human generated summary types. The McNemar test resulted in a non-significant *p*-value of 0.289 at the 5% significance level, and the independent chi-squared test also produced non-significant test results (*χ*^2^ = 1.485, *p* = 0.223) at the 5% significance level. These cross-tabulation test statistics can be seen in Table 4. Therefore, the present study fails to support comprehension results being dependent on summary type. Since the comprehension test results were not found to be dependent on the summary type being AI or human generated, we infer that the quality of the content retained within the summaries was not significantly different, and that the quality of the article summary types was comparable.

**Table 4:** Analysis of cross-tabulation data, comparing correct and incorrect comprehension test frequencies between AI and human summary groups, including the paired chi-squared McNemar test.

2×2 Cross-tabulation analyses				
	Value	df	Asymptomatic significance (two-sided)	Exact significance (two-sided)
Pearson Chi-Square	1.485	1	0.223	
Continuity correction	1.107	1	0.293	
Likelihood ratio	1.474	1	0.225	
McNemar test				0.289
N of valid cases	171			

## Discussion

While various AI tools exist, such as Bing Chat and Bidirectional Encoder Representations from Transformers (BERT), this study focused on GPT-3 by OpenAI (Ray, 2023). GPT-3 was selected due to its public accessibility that utilizes large datasets to create human-style text. Additionally, restricting the study to one tool helped to limit participant fatigue and maintain engagement.

The results of this study agree with remarkably similar work done by Hakam et al. (2024), which also compared qualities of AI-generated and human generated summaries of medical research, but within orthopedic academic literature specifically. Their study also found no significant differences between summary types, and readers could not differentiate summary types. The present study’s inability to reject the null hypothesis may indicate that medical students found no true discernable difference between the texts. While possible, it could also be possible that the present study’s participant pool assumed all summaries were similar in readability based on preconceived notions of students working toward medical degrees, since the original instructions to participants were that the summaries could be generated by MD, DO, and PA students. A future study might not deceive participants in this way, to avoid bias based on assumptions about the summary sources. Hakam et al. (2024) informed participants of the potential for AI-generated summaries, which may change participant expectations in its own way by introducing bias against AI systems. Thus, while managing participants’ bias toward artificial intelligence proves difficult, both methods of informing and deceiving participants may provide unique benefits worth exploring.

The present results, and the results from Hakam et al. (2024) draw slightly different conclusions compared to Gao et al. (2023), which compared new scientific abstracts generated by ChatGPT to original abstracts. Gao et al. (2023) found that blinded human reviewers successfully detected 68% of generated abstracts as being from ChatGPT, while 14% of original abstracts were falsely stated to be from AI. Despite the moderate success of blinded human reviewers in this case, the reviewers also indicated surprising difficulty in the task, while noting that AI-generated abstracts seemed vaguer and more formulaic than original abstracts. Similarly, Pinto et al. (2024) compared a ChatGPT written case report to that of a medical professional with 10 years of experience and found that only 12 of 22 reviewers correctly identified the AI-generated manuscript while 4 of 22 incorrectly identified the manuscript. They also found that the human manuscript was rated higher for quality of presentation and nuanced writing. Liao et al. (2023) concluded that human generated medical literature was more concrete and diverse, while AI-generated content paid more attention to being fluent and logical. Similarly, Mostafapour et al. (2024) compared a human-generated literature review with an AI-literature review using GPT-4 and found that while AI-generated text demonstrated diversity in knowledge, the text showed limitations in depth and contextual understanding. They also found that AI-text contained more incorrect and irrelevant information. Collectively, while many studies agree with the present study in suggesting that AI-text can reach levels of fluency and readability equal to human text, differences may exist in the depth, nuance, context, and most importantly factuality of the content. AI-generated literature must strive to not only be fluent and grammatically correct, but to be factual, evidence-based.

Considering limitations reflective of our participant population, the present study participants were medical students participating in a sleep medicine conference. These students were possibly more informed about sleep medicine compared to other medical students and hence create user bias. These students may be better or worse judges of literary readability based on their experience with sleep medicine, medical research, and writing. Consequently, they may comprehend the summaries based on knowledge of sleep medicine, resulting in the frequency of correct and incorrect answers in the comprehension test being biased as well.

Another limitation in the present study is that only three articles were used in total, so the specific articles’ content or difficulty may reduce generalizability to other research studies and medical fields. Future research including more sleep medicine articles, and more medical articles from different medical domains would improve the generalizability of the tested concepts regarding the ability of AI systems to summarize medical literature.

Data lacks demographic information. It would be helpful to know gender based differences in data.

Lastly, all participants came from a medical school sleep medicine conference, so there could be biased sleep medicine knowledge overall compared to the average medical student. This could have inflated all comprehension scores, since all three articles were topics in sleep medicine, obscuring true differences in summary quality. While this study focused on participants from a specific medical field, future research should include students and professionals from a broader range of medical disciplines, such as nursing, therapy, and other allied health professions, to enhance the generalizability of the findings.

Regarding methodological limitations in this study, the order of the articles in each form was not randomized, so there could be an order effect affecting both Form A and B. Additionally, the three comprehension questions and their answers were not randomized between forms, so there could have been a resultant order effect from the individual questions or answers. Also, participants answered multiple comprehension and readability questions from the AI summary and human summary categories, rather than the categories being tested in completely independent participant groups, which may impact test results.

Some of the most glaring drawbacks in replacing human generated science writing with AI-generated writing are the potential for inaccuracies and misinformation. Beyond readability and content quality, AI-generated medical summaries in clinical settings are not only assessed for their accuracy, but for potential clinical risks they may pose as well (Tang et al., 2023). Evaluating metrics of AI-generated content is essential, as factual authenticity avoids misinterpretations that could lead to misleading conclusions, impacting patient care. Human mistakes and limitations in medical research may not be considered or prioritized when AI tools are tasked with summarizing medical information, thereby propagating misinformation, and promoting research with limitations. Ma et al. (2023) found that AI systems were more likely to produce factual errors compared to human written content, and Athaluri et al. (2023) describes how AI systems can create false content when tasked to retrieve information from published medical research, sometimes called AI “hallucinations.” Furthermore, Salvagno et al. (2023) warns of the risk of plagiarism by AI-generated medical writing. Given the infancy of the technology’s applications, despite its rapid advancement and successes in recent years, AI models rely on the data used to train the model, while nuance and accountability by AI may be limited. In fact, many publishers like *Springer Nature* and groups like the International Committee of Medical Journal Editors and the Committee on Publication Ethics currently do not accept AI models as satisfying authorship criteria due to the lack of accountability for AI-generated work (Sallam, 2023; Committee on Publication Ethics, 2023). Summaries from these AI generated articles could potentially increase misinformation and misguide users who lack adequate experience. And even if medical journals do eventually accept primarily AI-generated content as publishable due to more advanced and validated models, Elali and Rachid (2023) warn of the total falsification of medical research by using AI-generated content. With these ethical considerations in mind, Dergaa et al. (2023) and Gaggioli (2023) both strongly recommend that authors must be transparent in their use of AI systems in medical writing. *Springer* Nature (2023) also calls for transparency by requiring disclosure of the use of AI systems in papers’ methods or introduction sections. On the other hand, Mavrogenis and Scarlat (2023) discuss the beneficial potential for AI models to generate synthetic data and medical research as promising opportunities for advancing medicine. Benichou and ChatGPT (2023) also conclude that AI models should be considered as tools to produce higher quality medical research more quickly. Collectively, it becomes clear that AI and LLMs must be evaluated rigorously as they become incorporated into academic research and medical education, and that transparency should be required by journals and educational programs for maintaining ethical standards. We agree with Gao et al. (2023) in supporting the implementation of AI-output detector applications as editorial tools by journals, while acknowledging that this requires improvement of the sensitivity and specificity of these tools. Teixeira da Silva (2023) also calls upon editors, journals, and publishers as being responsible for detecting AI content in literature. There remains a shared responsibility to ensure academic integrity and transparency in published scientific literature.

AI-derived inaccuracies may be particularly dangerous in medical research that lacks rigorous peer reviewing, and in medical domains that may be neglected or rushed in both medical education and clinical practice. For example, Romiszewski et al. (2020) described how topics in sleep medicine are often neglected, incompletely taught to medical students, and subsumed by other specialties in clinical practice. As a result, relying on incomplete or misinformed AI-generated content may impact physician and patient education in these fields. The spread of misinformation or incomplete information further by AI-generated content may reduce knowledge of evidence-based ideas in these vulnerable medical domains, which could have a collective impact on public health agendas addressing these vulnerable medical domains like sleep. As a result, while utilizing AI-generated content, physicians and medical students must not depend on the tools. Rather, AI-generated summaries of existing medical information should be used as an accessory form of learning before more rigorous investigation and fact-checking. AI models summarizing medical information should also provide citations, and individuals or authors should review cited works against the generative AI content before clinical or educational implementation.

Future directions for improving the present study’s objectives regarding AI systems and their ability to summarize sleep medicine research, or other forms of medical research, include using AI to summarize more types of research outside of sleep medicine to improve the generalizability of this capacity of AI in other fields. In addition to expanding across more fields of research, more types of publications should be studied, to assess the ability of AI to process varying article types like experimental studies, cross-sectional studies, prospective studies, randomized controlled trials, literature reviews, meta-analyses, and case studies. Furthermore, analyses could be done on the ability of AI to process and summarize specific aspects of articles, like pathophysiology, pharmacology, or data analysis components. Research should also be done on multiple types of AI models, as these systems are being produced by multiple private entities.

Despite the risks, great utility exists in using AI models to improve patient and physician education. As medical research is rapidly produced, the immense capacity of AI systems to process substantial amounts of information could improve exposure to new research for many. Korteling et al. (2021) described how AI systems process and generate information in a fraction of the time compared to humans, which could accelerate the spread of information, create more time for peer discussion, and increase the production of effective medical teaching materials at varying comprehension levels for physicians, students, and patients. If AI models can be validated in collecting and distributing comprehensive, well-translated medical information, immense value exists for distributing new medical research quickly after publication in the form of readers digests. Scalable tools like AI-generated readers digests could be used for students, patients, and physicians. Furthermore, online tools like AI-generated readers digests of medical research could enable users to toggle settings that change aspects of the underlying prompt, to focus the AI-generated content on specific aspects of the research like data, methodology, or results. This would not only create transparency regarding the content presented, but it would also allow users of AI-driven tools to have more control over the information shown.

To reduce errors in AI-generated content, curators of these readers digests and of AI-generated content in general should employ a hybrid model whenever possible, in which humans help to proofread AI-processed content. Bellini et al. (2023) foresees this AI-human hybrid model as a standard paradigm in the future, representing an inevitable progression within healthcare digitalization. For example, when submitting an article to a journal, the submission process could use AI to generate article summaries, and the journal could have both authors and editors validate the summary for future use in a reader’s digest.

With more rapid exposure to new research, medical research can be analyzed and built upon more quickly, which may improve healthcare outcomes through improved physician education and patient literacy. Lastly, AI systems and readers digests could save time for physicians, thereby improving provider quality of life, reducing burnout, and granting more time to focus on the patient during clinical visits. These benefits may prove to be particularly beneficial for domains like sleep medicine, in which exposure to new research for medical professionals and even the public may provide clinically useful, practical benefits.

## Data Availability

The raw data supporting the conclusions of this article will be made available by the authors, without undue reservation.
